# SURF1 knockout cloned pigs: Early onset of a severe lethal phenotype

**DOI:** 10.1016/j.bbadis.2018.03.021

**Published:** 2018-06

**Authors:** C. Quadalti, D. Brunetti, I. Lagutina, R. Duchi, A. Perota, G. Lazzari, R. Cerutti, I. Di Meo, M. Johnson, E. Bottani, P. Crociara, C. Corona, S. Grifoni, V. Tiranti, E. Fernandez-Vizarra, A.J. Robinson, C. Viscomi, C. Casalone, M. Zeviani, C. Galli

**Affiliations:** a Avantea, Laboratory of Reproductive Technologies, Via Porcellasco 7/f, Cremona 26100, Italy; b University of Cambridge/MRC Mitochondrial Biology Unit, Wellcome Trust/MRC Building, Hills Rd, Cambridge CB20XY, UK; c Fondazione Avantea, Cremona, Italy; d Istituto Zooprofilattico Sperimentale del Piemonte Liguria e Valle d'Aosta, Via Bologna 148, Torino 10154, Italy; e Dept. of Veterinary Medical Sciences, University of Bologna, Via Tolara di Sopra 50, 40064 Ozzano dell'Emilia, BO, Italy; f Neurologic Institute Carlo Besta, Via G. Celoria 11, 20133 Milan, Italy

**Keywords:** Leigh syndrome, SURF1 KO, Genome editing, Pig, Mitochondrial disease

## Abstract

Leigh syndrome (LS) associated with cytochrome *c* oxidase (COX) deficiency is an early onset, fatal mitochondrial encephalopathy, leading to multiple neurological failure and eventually death, usually in the first decade of life. Mutations in *SURF1*, a nuclear gene encoding a mitochondrial protein involved in COX assembly, are among the most common causes of LS. LS^SURF1^ patients display severe, isolated COX deficiency in all tissues, including cultured fibroblasts and skeletal muscle. Recombinant, constitutive *SURF1*^*−/−*^ mice show diffuse COX deficiency, but fail to recapitulate the severity of the human clinical phenotype. Pigs are an attractive alternative model for human diseases, because of their size, as well as metabolic, physiological and genetic similarity to humans. Here, we determined the complete sequence of the swine *SURF1* gene, disrupted it in pig primary fibroblast cell lines using both TALENs and CRISPR/Cas9 genome editing systems, before finally generating *SURF1*^−*/*−^ and *SURF1*^*−/+*^ pigs by Somatic Cell Nuclear Transfer (SCNT). *SURF1*^*−/−*^ pigs were characterized by failure to thrive, muscle weakness and highly reduced life span with elevated perinatal mortality, compared to heterozygous *SURF1*^*−/+*^ and wild type littermates. Surprisingly, no obvious COX deficiency was detected in *SURF1*^*−/−*^ tissues, although histochemical analysis revealed the presence of COX deficiency in jejunum villi and total mRNA sequencing (RNAseq) showed that several COX subunit-encoding genes were significantly down-regulated in *SURF1*^*−/−*^ skeletal muscles. In addition, neuropathological findings, indicated a delay in central nervous system development of newborn *SURF1*^*−/−*^ piglets. Our results suggest a broader role of sSURF1 in mitochondrial bioenergetics.

## Highlights

• The full sequence of pig *SURF1* gene was determined.
• *SURF1* gene was disrupted in pig by gene editing and somatic cell nuclear transfer.
• *SURF1*^*−/−*^ piglets showed an early onset lethal phenotype.
• Mitochondrial bioenergetics was impaired in the skeletal muscle of *SURF1*^*−/−*^ pigs.

## 1 Introduction

Leigh syndrome (LS, OMIM 256000) associated with cytochrome *c* oxidase (COX) deficiency is an early onset, genetically heterogeneous disease characterized by subacute neurodegenerative encephalopathy [1,2]. The typical course of LS includes neurodevelopmental regression, brain stem and basal ganglia signs (ataxia, dystonia, chorea, optic atrophy, ophthalmoparesis, swallowing and/or feeding difficulties, tongue fasciculations, and apnoeic episodes) and characteristic magnetic resonance imaging (MRI) findings, including symmetrical lesions in the basal ganglia, thalamus, cerebellum, and brain stem. Other manifestations may include failure to thrive, microcephaly, hypertrichosis, and myopathy [1,3,4]. Lactic acid levels in the blood and cerebrospinal fluid of the patients are often elevated, and COX activity is <20% compared to normal fibroblasts, lymphocytes, or muscle biopsies. COX is the terminal component of the mitochondrial respiratory chain and catalyzes the electron transfer from reduced cytochrome *c* to oxygen whilst pumping protons from the mitochondrial matrix to the intermembrane space, across the inner mitochondrial membrane.

Mutations in *SURF1* account for about one third of LS cases and are by far the most frequent cause of LS associated with isolated COX deficiency [5,6]. Although *SURF1*-related LS is a well-established neuropathological and clinical entity, atypical manifestations have been occasionally reported, such as long-surviving cases with scarce brain lesions [3,7], or subjects manifesting predominant demyelinating Charcot-Marie-Tooth disease [8], villous atrophy, hypertrichosis without the typical brain lesions [9], or severe renal involvement [10]. Some cases of leukodystrophy with systemic COX deficiency were also reported [3,11,12]. However, severe isolated COX deficiency and necrotizing encephalomyelopathy remain the most common biochemical and clinical-pathological hallmarks of the disease.

In humans, the *SURF1* gene is located on chromosome 9q34 in a cluster of genes with a peculiar genomic structure conserved throughout the vertebrate radiation, called the *surfeit locus*. Orthologues of SURF1 are present in virtually all eukaryotes, from yeast to mammals. Human SURF1 (hSURF1) is a 300-amino acid protein located in the inner mitochondrial membrane, deemed to be involved in the assembly and maintenance of COX, although its exact function is still not fully understood. Truncating mutations in the N-terminal, central, or C-terminal regions of hSURF1 result in an unstable protein unable to rescue COX activity in SURF1-deficient cell lines [13]. In the last decade, several attempts to generate SURF1-deficient LS animal models have been made, with controversial results. For instance, constitutive recombinant *SURF1*^*−/−*^ mice showed mild but significant COX deficiency and slight elevation of blood lactate, but failed to recapitulate the main human clinical signs, and yet they displayed a surprising increase in longevity and enhanced memory [14,15]. A SURF1 knock-down zebrafish model showed COX deficiency with cardiac and endodermal developmental defects, abnormal swimming behaviour, and increased apoptosis in the hindbrain and neural tube [16]. In a LS Drosophila model, the constitutive *SURF1* knockdown is lethal in larvae that however display defects in all complexes of the mitochondrial respiratory chain (MRC) and impaired muscular development. In a conditional central nervous system (CNS)-restricted Drosophila model the knockdown of *SURF1* leads to isolated COX deficiency in adult flies [17]. The failure of small animal models, in particular rodents, to mimic the main features of the human syndrome prompted us to generate a large animal model [18]. Over the last 20 years, swine (*Sus scrofa*) has been increasingly used as a suitable animal model in various biomedical research programs [[19], [20], [21]]. Pigs share a number of anatomic and physiologic features with humans that make them a promising animal model for investigating a range of human diseases. New genome-editing technologies, based on TAL effector-like nucleases (TALENs) and clustered regularly interspaced short palindromic repeats (CRISPRs)/Cas9 site-specific nucleases (SSNs), recently boosted the use in research of species other than mouse. Here we exploited these technologies to generate a *SURF1*^*−/−*^ pig, the first swine model of a mitochondrial disease.

## 2 Materials and methods

### 2.1 Animal experiments

All procedures involving the use of animals in this study were approved by the Local Ethics Committee of Avantea, carried out in accordance with the Italian Law (D.Lgs 26/2014) and EU directive 2010/63/EU regulating animal experimentation and approved by relevant authorities (Ministry of Health project n 606/2016-PR).

### 2.2 Chemicals

All chemicals and reagents were purchased from Sigma (Milan, Italy), unless otherwise stated.

### 2.3 Amplification and sequencing of the swine genomic *SURF1* gene

The whole *sSURF1* gene was PCR-amplified using S1F-long and S1R-long primers using genomic DNA of cell line ID6639 (commercial pig hybrid, Large White x Landrace) as a template. The sequences of all the primers used in this study are reported in Supplementary Table S3. Amplification was performed in 12.5 μL reaction volume (0.4 mM dNTPs, 0.8 μM each primer, 0.05 U/μl LA-Taq in GCI Buffer, Nuclease-free H_2_O to volume) using a touchdown protocol (Supplementary methods).

The PCR product (4582 bp, Supplementary Fig. 1A) was cloned into TOPO-TA vector (Invitrogen, Thermo Scientific, Waltham, MA USA), transformed into competent DH5α strain of *E. coli* and Sanger-sequenced (GATC Biotech, Constance, Germany). sSURF1 cDNA was cloned from the cell line ID6639 by reverse transcription of purified mRNA, followed by PCR amplification using gene specific primers S1F and S1R (Supplementary Table S3). RNA extraction was performed using a guanidine thiocyanate protocol [22]. Reverse transcription was performed using RevertAid H Minus First Strand cDNA Synthesis Kit (Thermo Scientific, Waltham, MA USA). The specific PCR product is 921 bp. This cDNA sequence was used to confirm the sequences of exons 1 to 9.

### 2.4 Cell isolation and culture

Primary porcine fibroblast cultures were derived from adult Large White × Landrace hybrid female (ID167, Avantea) and male (ID6639) ear biopsies (pig adult fibroblasts, PAFs) and 40-day-old male foetus biopsy (Pig Foetal Fibroblasts after CRISPR editing, PFFs; IDclone-5, cloned from ID6639), by cutting the biopsies in small pieces with a scalpel blade. Tissue pieces were plated in triplicate in 60-mm culture dishes with 2 mL of DMEM/TCM199 (1:1) supplemented with 20% Foetal Bovine Serum (FBS). Cells were allowed to grow until 50% of confluence with medium changes every 3 days, then tissue fragments were removed and cells sub-cultured in 4 mL of DMEM/TCM199 (1:1) supplemented with 10% FBS and bFGF (5 ng/mL) until 80% of confluence. Cells were then cryopreserved at early passages in DMEM/TCM199 (1:1) with 20% FBS and 10% DMSO and stored in liquid nitrogen. These batches were used throughout the experiments. The same procedure was followed for the setting up of SURF1 animals cell lines bio-bank.

### 2.5 CRISPR/Cas9 and TAL effector nucleases (TALENs) design and validation

CRISPR/Cas9 system sgRNA guides were designed to target swine SURF1 exon 3 according to the software CRISPR Design Tool (www.crsipr.mit.edu). Two high quality guides, with predicted high efficiency (score > 50) were chosen (Supplementary Table S2). Complementary oligonucleotides for each guide were annealed to generate duplex and cloned in the CAG-hspCas9-H1-gRNA linearized Smart Nuclease vector according with the user manual (CAS920A-1 System Biosciences).

TALEN vectors design and production was outsourced to Genecopoeia (www.genecopoeia.com) to target exon 3 and exon 4 of the swine SURF1 gene (GenBank: AK391958.1). Three different TALENs pairs were purchased (Supplementary Table S1). For each TALEN pair, pig fibroblast (1 ∗ 10^6^ cells, ID6639) were transfected (Nucleofector TM, LONZA, Basel, Switzerland) and each pool obtained was plated in 60-mm culture dish and harvested 72 h post transfection for lysis and genomic DNA extraction. Both CRISPR/Cas9 and TALENs efficiency was evaluated through Surveyor® Assay (IDT Surveyor® Mutation Detection Kit, Iowa, USA).

### 2.6 Transfection of pig fibroblasts and screening

Initial transfection with CRISPR/Cas9 were performed without the targeting vector and puromycin selection generated only *SURF1*^*+/−*^ cells, therefore a two-step strategy was implemented to obtain *SURF1*^*−/−*^ fibroblasts (Supplementary Fig. 3). As a consequence for the TALENs editing strategy, aiming at the one step generation of *SURF1*^*−/−*^ fibroblasts the targeting vector was always used to generate both males and females edited cells.

PAFs were thawed two days before transfection in order to have 1 ∗ 10^6^ cells/transfection.

For CRISPRs transfection experiments, cells were prepared as above and Nucleofector TM solution was supplemented with 3 μg of the CAG-hspCas9-H1-gRNA vector with or w/o the addition of 2 μg of the HR vector (1:1) for co-transfection experiments. The cell suspension was transferred in the transfection cuvette and program V-024 was used for all transfections, as previously described [23]. For colony isolation, transfected cells were plated single-cell in 150-mm culture dishes with fresh culture medium. Where HR vector was used, after 24 hours puromycin (1 μg/mL) was added for selection. After 8 days of antibiotic selection, puromycin-resistant colonies were picked and transferred into 24-well dishes with fresh culture medium. For each colony, an aliquot was cryopreserved in liquid nitrogen (DMEM/TCM199 1:1, 20% FBS and 10% DMSO) for subsequent SCNT (Somatic Cell Nuclear Transfer) and the remaining cells lysed for genomic extraction, PCR-analysed and Sanger-sequenced for both the integration of the targeting vector and the presence of InDels. Genomic DNA was extracted (see Supplementary methods), amplified with the primers Ex2Fw – Ex4Rv (see Supplementary Table S3) and sequenced. Confirmed NHEJ-mutated *SURF1*^*+/−*^ colonies were used for first step-SCNT and foetuses (D + 40 of gestation) were collected to establish PFF cell lines. The rejuvenated PFF genotype was confirmed and cells were used for a second round of co-transfection (HR vector + CAG-hspCas9-H1-gRNA 1:1) to obtain *SURF1*^*−/−*^ colonies. Puromycin resistant colonies were screened by PCR using Ex2Fw and PuroF2-R oligos (Supplementary Table S3). First-step *SURF1*^*+/−*^ NHEJ-mutated colonies were also used as nuclear donor cells for the generation of *SURF1*^*+/−*^ piglets.

For TALENs transfections, cells were trypsinized, counted and resuspended in Nucleofector TM solution (Basic Nucleofector TM Kit, Primary Fibroblasts, LONZA, Basel, Switzerland) supplemented with 2 μg of each TALEN arm (L + R) plasmid and 1 μg of HR vector (not used in transient transfection) (Supplementary methods). Recombinant colonies were screened by PCR for NHEJ-derived mutations using the S1TF and S1TR oligos giving a 375 bp product (Supplementary Table S3); for the integration of the HR vector the Ex2F + PuroF2-R oligos, amplifying a 1237 bp fragment, were used (Supplementary Table S3). NHEJ was confirmed by Sanger sequencing (Supplementary methods).

### 2.7 Somatic Cell Nuclear Transfer (SCNT)

The protocol used was the one described in [24] with minor modifications. Briefly, ovaries of pubertal gilts were collected at a local abattoir and transported to the laboratory at 31 °C–33 °C. Follicles larger than 3 mm were aspirated and cumulus-oocyte complexes were selected and in vitro matured in defined maturation medium at 38.5 °C in 5% CO_2_ in humidified air for 42 h. The day before SCNT, selected nuclear donor cells were induced into quiescence by serum starvation (0.5% FCS). The day of SCNT, cells were trypsinized, washed and resuspended in SOF [25] supplemented with 25 mM Hepes (SOF-Hepes). At the end of maturation process, only matured oocytes were selected (presence of an extruded polar body). NT-embryos were reconstructed following a zona-free method [26], where the zona pellucida was digested with 0.5% pronase in PBS. All manipulations were performed in SOF-Hepes with 10% FCS. Enucleation was performed following a 5 minutes exposure to cytochalasin B (7.5 μg/mL) and Hoechst (5 μg/mL). Enucleated cytoplasts were then rinsed in phytohemagglutinin P in PBS and quickly dropped over a single nuclear donor cell prepared after trypsinization. Donor cell-cytoplast couples were washed in 0.3 M mannitol solution and fused by double DC-pulse (1.2 KVolt/cm) and returned in to maturation medium. After 2 h, at about 48 h of maturation, NT embryos were activated by double DC-pulses [24], followed by 2 h in maturation medium supplemented with cytochalasin B (5 μg/mL) and cycloheximide (10 μg/mL) to avoid polar body extrusion. At the end of the activation, reconstructed embryos were cultured in mSOF supplemented with essential and non-essential amino acids and with 4 mg/mL BSA, in a modification of the Well Of the Well system [27] and the culture was continued as described in [24].

### 2.8 Recipient sows synchronization, surgical Embryo Transfer (ET) and post-implantation development

Recipient sows oestrus was synchronized by feeding 12 mg of altrenogest (Regumate, Intervet, Peschiera Borromeo, Italy) per sow for 15 days, followed by an injection of 0.15 mg of PgF2α (Dalmazin, Fatro, Ozzano Emilia, Italy) at the 13th day of altrenogest treatment and one of 1000 IU of hCG (Chorulon, Intervet, Peschiera Borromeo, Italy) 96 h after the last altrenogest treatment. SCNT embryos were surgically transferred to the uterus of synchronized sows on day 6 of development (compacted morula and early blastocyst stages) through a mid-ventral laparotomy procedure [28], five days after standing oestrus. Recipients were checked for pregnancy by trans-abdominal ultrasound examination 30 days after ET and pregnancy were confirmed around day 60 post-ET. Farrowing was induced by a PgF2α injection on day 119 and 120 of gestation. After birth, all animals were genotyped (Supplementary methods).

### 2.9 Isolation of mitochondria

Standard methods were used for the preparation of mitochondrial and post-mitochondrial fractions from pig tissues (brain, muscle and liver) [29] (Supplementary methods).

### 2.10 Biochemical and clinical analysis

Spectrophotometric assays of individual respiratory complex activities were carried out on tissue homogenates and on cultured cell lysates as described [30]. Specific activities of each complex were normalized to that of citrate synthase (CS), an index of mitochondrial mass. Blood lactate was measured using the Lactate Assay Kit and protocol (Sigma, St Louis, MO, USA). For newborns, the Lactate Scout Analyzer [31] (EKF Diagnostics, Cardiff) was used in order to minimize the amount of blood, and thus stress. Measurements started a few hours after birth and continued periodically along the piglets' life. As piglets were allowed to feed under the recipient mother sow and differences in birth weight can influence their capacity to feed properly in competition with littermates [32], we divided animals in weight groups and compared, for each group, *SURF1*^*−/−*^ and *SURF1*^*+/−*^ animals, and age- and weight-matched controls. We also measured blood lactate levels in one longer-lived *SURF1*^*−/−*^ animal (ID498 at 58 days of age) compared with two age-matched controls. In order to avoid side effects related to pad puncture of heavier animals, we chose to take a 200 μL blood sample from ear vein and measure blood lactate following the instructions of the Lactate reagent kit (Sigma); we followed this procedure for both ID498 and relative controls.

Standard blood test analyses were performed on *SURF1*^*−/−*^, *SURF1*^*+/−*^ and age-matched control animals. In addition to standard blood analysis, TSH, FT3 and FT4 levels were also analysed using standard diagnostic methods.

Western blot, Blue Native and In-Gel activity analysis are described in Supplementary methods.

### 2.11 Histochemical and histological analysis of pig tissues

For histochemical studies of quadriceps and jejunum samples, tissues were frozen in liquid-nitrogen precooled isopentane and serial 8-μm-thick sections were stained for COX, SDH and NADH as previously described [33].

Brains were fixed by immersion in 10% buffered formalin, embedded in paraffin and 5 μm sectioned. For histological analyses, sections were stained with hematoxylin–eosin or Luxol Fast Blue/Hematoxylin and visualized under a light microscope (Leica Microsystem). For immunohistochemistry, sections were quenched for 30 min in methanol and 0,3% hydrogen peroxide. Antigen retrieval was performed by incubating the slides in 10 mM citrate buffer (pH 6.0) for 15 min. For immunofluorescence analyses, brains were fixed in 4% PFA, cryoprotected in 30% sucrose and embedded in O.C.T. compound frozen in liquid-nitrogen-cooled isopentane. Twenty μm thick sections were cut with cryostat, permeabilized in 0,5% triton X- 100 in PBS for 20 min and blocked in 5% normal donkey serum in PBS for 1 h. The following primary antibodies were incubated overnight at 4 °C: anti-GFAP (1: 1000; Millipore), anti Iba 1 (1: 600, Biocare), anti-Cleaved Caspase 3A (1: 300, Cell Signaling), anti MBP (1: 400, Millipore), anti DCX (1:1000, abcam).

After several washes, slides were incubated with the biotinylated secondary antibody followed by ABC complex reagents (Vector Labs) and DAB staining. For immunofluorescence, the following secondary antibodies were used: donkey Alexa Fluor 488 or 555 conjugated (Thermo Fisher Scientific). Slides were then washed in PBS, counterstained with DAPI and mounted with Fluoromount G (SouthernBiotech). All fluorescence images were captured on a confocal laser- scanning microscope (Leica TCS SP8, Leica Microsystem). To label endothelial cells biotinylated Griffonia Simplicifolia Lectin I (Isolectin B4 1: 400, Vector Labs) was used.

### 2.12 Quantification of brain sections

The thickness of the cerebral cortex grey matter was quantified using the software NIS Elements (Nikon). Object identification was performed using a protocol adapted from Lin et al., 2005 and Chen et al., 2007 [34,35]. A constant sized image segment was converted to 8 bits per pixel grey scale. The images were then binarized using a fixed interval intensity threshold series, followed by erosion/dilation filtering to detect boundaries of object. Fluorescent signal areas were binarized, quantified and expressed as a percentage of the total area. Image analyses were performed using ImageJ.

### 2.13 Mitochondrial morphology evaluation

Mitochondrial morphology was assessed after vital cell staining with 10 nM Mitotracker CMX-Red (Invitrogen) for 30 min at 37 °C. Fluorescence was visualized with a digital imaging system using an inverted epifluorescence microscope with a 63/1.4 oil objective (Nikon, Japan). Images were captured with a back-illuminated Photometrics Cascade CCD camera system (Crisel).

### 2.14 - Total mRNA sequencing experiment and data analysis

Differential expression of genes between the SURF1 knock-out and wild type samples was evaluated by DESeq2 [25516281] by IGA Technology Services, Udine, Italy. Quadriceps samples from 2 live *SURF1*^*−/−*^ male animals on day 1 after birth and from 2 matching controls were collected immediately after euthanization and conserved at −80 °C in RNAlater. TruSeq Stranded mRNA Sample Prep kit (Illumina, San Diego, CA) has been used for library preparation following the manufacturer's instructions, starting with 1–2 μg of good quality RNA (R.I.N. >7) as input. The poly-A mRNA was melted 3 min at 94 °C and every purification step has been performed by using 1× Agencourt AMPure XP beads. Both RNA samples and final libraries were quantified by using the Qubit 2.0 Fluorometer (Invitrogen, Carlsbad, CA) and quality tested by Agilent 2100 Bioanalyzer RNA Nano assay (Agilent technologies, Santa Clara, CA). Libraries were then processed with Illumina cBot for cluster generation on the flowcell, following the manufacturer's instructions and sequenced on paired-end 125 bp mode on HiSeq2500 (Illumina, San Diego, CA) with a median 73.8 M of reads/sample (min 52.8 M, max 86.3 M). The CASAVA 1.8.2 version of the Illumina pipeline was used to process raw data for both format conversion and de-multiplexing.

Statistically significant enrichment of GO terms in genes that were under- or over-expressed was evaluated by using DAVID [PMID: 22543366].

### 2.15 Statistical analysis

Two-tailed, unpaired, unequal variance Student's *t*-test and ANOVA with post-hoc Tukey HSD test were used for statistical analysis. Survival probability was calculated using the Kaplan–Meier and log-rank tests (XLSTAT, Addinsoft). The data are shown as mean ± SEM.

For RNAseq analysis, we determined whether the biases in the expression levels were statistically significant.

## 3 Results

### 3.1 Molecular characterization of the swine SURF1 gene

**Fig. 1:**
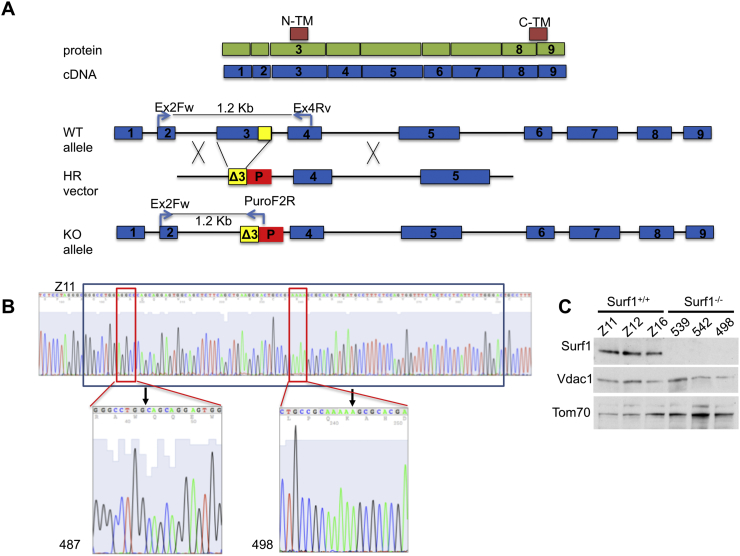
Schematic representation of experimental designs for the generation of SURF1^−/−^ cells and derived pigs. A) Schematic representation of SURF1 protein and cDNA, and of the strategy used to generate the mutant allele with the targeting vector. N-TM and C-TM, N- and C-terminal transmembrane domains, HR, homologous recombination; P, puromycin resistance cassette; Ex, exon. Ex2Fw, Ex4Rv, and PuroF2R: oligos used to PCR amplify the genomic *SURF1* alleles, giving the bands indicated. Δ3, in yellow is the residual exon 3 after the insertion of the targeting vector. B) NHEJ of the second allele. Sequence of a *SURF1*^*+/+*^ (Z11; upper sequence) and cloned *SURF1*^*−/−*^ pigs (lower panels). The black box in the wild-type allele indicates the 106 bp sequence eliminated by homologous recombination in the targeted allele. The red box on the left part of the wild type sequence indicates the position of the 5-bp deletion obtained using TALENS (#487); the sequence of the mutant allele is magnified below. The black arrow indicates the junction point. The red box on the right part of the wild type sequence indicates the region where an additional A (black arrow) has been inserted by CRISPR/Cas9; the sequence of the mutant allele is magnified below (#498). These results demonstrate that cloned *SURF1*^*−/−*^ individuals carry one allele disrupted by the insertion of Puromycin and the other allele containing either the 5 bp deletion obtained by TALENs (ID 487) or the 1 bp insertion obtained by CRISPR/Cas9 editing. As a result, cloned *SURF1*^*−/−*^ pigs contain two null *SURF1* alleles. C) WB on liver mitochondrial enriched fraction from 3 *SURF1*^*+/+*^ and a *SURF1*^*−/−*^ pigs, showing the absence of SURF1 protein in *SURF1*^*−/−*^ pigs. VDAC1 and TOM70 were used as loading controls. Pigs 539 and 542 were generated using TALENs, pig 498 CRISPR/Cas9.

Only three exons (exons 2–4) of swine SURF1 gene were annotated on public databases (NC_010449.4). We used this information and the human *SURF1* (*hSURF1*) sequence to PCR-amplify the full-length swine *sSURF1* genomic locus from the wild type cell line ID6639. A single PCR product of 4582 bp was obtained (Supplementary Fig. 1A) and fully sequenced (NCBI: BankIt2034014 Surf1 MF535518). By using the swine mRNA (NCBI AK391958) and the *hSURF1* genomic (NG_008477.1) sequences, we were able to place all 9 exons (Fig. 1A). The sequences of the exons were further validated by directly sequencing the *sSURF1* cDNA extracted from pig fibroblasts. The human and swine proteins showed 80% identity and 87% similarity by Clustal OMEGA alignment (Supplementary Fig. 1B). Further genomic analysis located the *sSURF1* gene on chromosome 1 (ENSSSCG00000035727).

### 3.2 Disruption of *SURF1* gene in swine primary fibroblasts

For the CRISPR/Cas9 experiment, we initially used a specific guide RNA (gRNA) to disrupt the *sSURF1* gene by non-homologous end joining (NHEJ) in the male cell line. We obtained 16/118 colonies with site-specific InDels, in particular all were characterized by a single base pair insertion (+A) (Table 1 and Supplementary Fig. 2B) and one of these (F4) was used for SCNT to obtain rejuvenated porcine foetal fibroblasts (PFFs, clone-5). These were then used to perform a second round of CRISPR/Cas9 transfection using the same gRNA in combination with the HR vector, which harbours a 106 bp deletion (Δ106) on the left homology arm of exon 3 as well as a puromycin-resistance (Puro-R) cassette, in order to obtain *sSURF1*^*−/−*^ colonies. We obtained 14/100 clones positive for the homologous insertion of the HR targeting vector. These were verified by PCR and Sanger sequencing, and four (171C2, 171C5, 171D4, 171F3) were used as nuclear donor cells in SCNT experiments. This two-step procedure is summarized in Supplementary Fig. 3. The functional outcome of this editing is the disruption of exon 3 on one allele by HR and of the other allele by the NHEJ-driven insertion of an A. The latter results in a frameshift mutation with a predicted mRNA product that will code for a protein of 64 aa against the original mRNA of 306 aa (see Fig. S1).

**Table 1:** Results of transfection experiments. Percentages are calculated on the total number of colonies picked up for each experiment. KO = SURF^−/−^; HET = SURF^+/−^. *colonies are not targeted (because the HR vector was not used in this transfection), but positive for the introduction of site-specific InDels.

Transfection mix	Fibroblast cell line ID (M/F)	N° colonies	N° targeted colonies (%)	N° sequenced colonies (%)	N° colonies suitable for SCNT (%)
Tal01L + R + HR vector	6639 (M)	48	32 (67%)	14 (29%)	10 (21%) KO
Tal02L + R + HR vector	6639 (M)	42	23 (55%)	8 (19%)	3 (7%) KO
Tal01L + R + HR vector	167 (F)	38	25 (66%)	5 (13%)	5 (13%) HET
CRISPR PAM90	6639 (M)	118	16 (14%)*	79 (67%)	10 (8%) HET
CRISPR PAM90 + HR vector	clone-5	100	14 (14%)	40 (40%)	10 (10%) KO

**Fig. 2:**
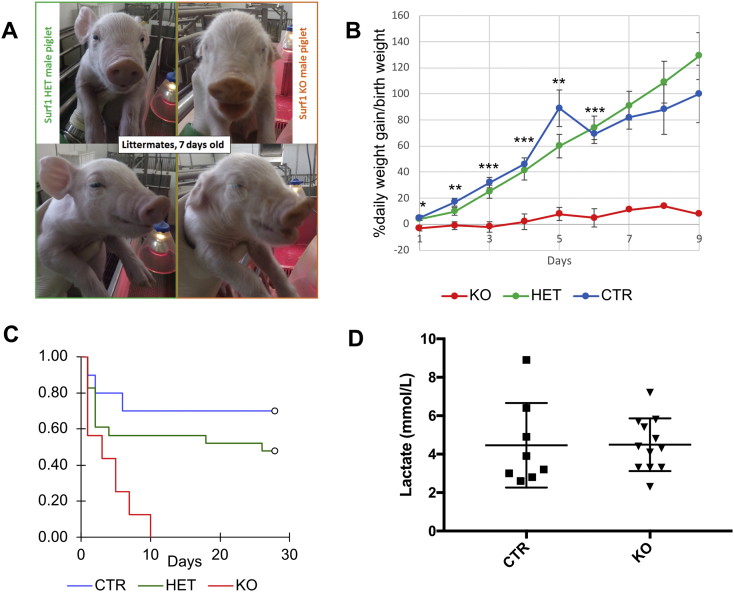
Phenotypic characterization of swine *SURF1*^*−/−*^ model. A) Pictures showing the facial dysmorphic features in *SURF1*^*−/−*^ compared to *SURF1*^*+/−*^ littermate piglets. B) Daily weight gain (% of birth weight) of *SURF1*^*−/−*^ (day 1, n = 20) vs. *SURF1*^*+/+*^ (day 1, n = 37) and *SURF1*^*+/−*^ (day 1, n = 28) piglets nursed under the mother during the first 9 days of life (animals artificially fed were excluded from this analysis). Data are presented as mean percentage of daily weight gain/birth weight ± SEM. The data were analysed using one-way ANOVA. The asterisks refer to the significance between *SURF1*^*−/−*^ and *SURF1*^*+/+*^ (CTR). *p<0.05; **p<0.01; ***p<0.005. No statistical analysis was carried out for ages 7–9 because only one *SURF1*^*−/−*^ animal per time point survived. C) Kaplan-Meier survival curves for the various genotypes. D) Blood lactate concentration in *SURF1*^*−/−*^ (KO; n = 12) vs. *SURF1*^*+/+*^ (CTR; n = 8) piglets at P1.

In the experiment with TALENs we obtained 32/48 and 23/42 targeted colonies with Tal01 and Tal02 respectively on the male cell line (Table 1). In both cases, the targeted clones carried the Puro-R targeting vector in one allele as a consequence of HR. We then verified the presence of additional mutations attributable to NHEJ in the second allele. Previous experiments showed that colonies carrying large (>20 bp) InDels were not suitable for SCNT and they were therefore discarded. Therefore 4 cell clones *sSURF1*^*−/−*^ carrying ≤5 bp, out-of-frame mutations were used for the first SCNT experiment: Tal01A6: ins. 4 bp (CAGC), Tal01C2: del. 5 bp (AGGCC), Tal01C7: del. 5 bp (AGCAG), Tal02B5: del. 2 bp (TC). We obtained 5 animals (see Fig. 2 first row) and then the apparently fittest animal, ID 487, obtained by Tal01C2: del. 5 bp (AGGCC), was used to re-clone by nuclear transfer all the animals of the study including those that were used for RNASeq (Supplementary Fig. 2). As in the case of CRISPR, also with TALENs these editing procedures resulted in the disruption by the insertion of Puro-R in exon 3 of one allele and in the NHEJ-driven 5 bp deletion in exon 3 of the other allele. The latter results in a frameshift mutation with a predicted mRNA product encoding a truncated protein of 53 aa against the wild-type one of 306 aa (see Fig. S1). The female cells were selected as described for the male cell line; 25/38 clones carried heterozygous mutations corresponding to the HR allele targeted with Puro-R and a WT allele. A pool of 6 of these clones (A2Tal01, A3Tal01, A6Tal01, B4TaL01, B5Tal01) was used for SCNT.

### 3.3 Generation of *sSURF1*^*−/−*^ and *sSURF1*^*+/−*^ pigs by SCNT

As for piglets deriving from TALENs experiments, we made a first SCNT experiment, using 4 cell clones with small Indel including the C2Tal01 clone and obtained a 5-piglet litter, four of which were born alive. In order to exclude potential large offspring syndrome, sometime occurring as a consequence of SCNT [36], we re-cloned one of these piglets (ID487, originated from C2Tal01) that showed no defects attributable to SCNT procedure to generate all the piglets for the subsequent experiments. In addition to genotypic validation, the *sSURF1*^*−/−*^ animals derived from ID487 recloning were phenotypically validated to lack the sSURF1 protein by Western-blot (WB) immunodetection (Fig. 1C).

A total of 24 SCNT experiments, resulting in 11 farrowings (TALENs- and CRISPR/Cas9-derived animals), were obtained, and a total of 75 animals of both sexes and all genotypes were generated (Table 2). SCNT efficiency ranged from 1% to 10%.

**Table 2:** Summary of cloned SURF1 piglets obtained throughout the SCNT experiments. All animals born were PCR-genotyped and the SURF1^+/−^ (HET) or SURF1^−/−^ (KO) condition was confirmed by Sanger sequencing. Taking into account the necessary genetic equivalence of piglets deriving from re-cloning experiments of ID487, only a representative random sample of piglets/litter were confirmed by Sanger sequencing.

Somatic cell nuclear donor line ID	M/F	KO or HET (nuclease + vector)	Offspring (% alive)	Genotype confirmed by Sanger sequencing
6639 (pig 487 delivery)	M	KO(TALENs + HR)	5 (80%)	4
487	M	KO (re-cloning)	32 (63%)	15
6639	M	KO (CRISPRs + HR)	1 (100%)	1
6639	M	HET (CRISPRs − NHEJ)	19 (74%)	19
167	F	HET (TALENs + HR)	18 (77%)	17

### 3.4 Clinical phenotype of *SURF1*^*−/*−^ piglets

Overall, 38 *sSURF1*^*−/−*^ male piglets were generated, 13 of which (34%) were stillborn. Eleven out of the 25 individuals born alive died or were culled the same day of birth due to severe clinical phenotype, including severe tremors, absent or weak suckling and rooting reflexes. Six *SURF1*^*−/−*^ piglets lived for 6–9 days and neurological examination revealed impaired hindlimb retraction and cranial tibial reflexes starting from day 3. These reflexes were normal in age-matched controls and *sSURF1*^*+/−*^ animals. In the following days, the *sSURF1*^*−/−*^ piglets developed failure to thrive and showed difficulties in swallowing with weak or absent suckling and rooting reflexes, inability to recognize food, poor weight gain (Fig. 2B) often associated with diarrhoea, and altered perception of the surrounding space with a tendency to hurt themselves. In two cases with farrowing of a single *sSURF1*^*−/−*^ piglet, the animals were removed from the sow and hand-raised in an intensive care unit under artificial feeding conditions. Under such conditions, the two piglets lived for more than one week (29 days and 80 days) compared to the piglets left nursing under the mother. Both piglets (one derived from CRISPR/Cas9-edited colony and one from TALENs-derived colony) showed facial dysmorphism (Fig. 2A), characterized by shorter nose, bilateral eyelid ptosis, weakness of facial muscles, in addition to pronounced body tremors with diffuse muscle weakness, respiratory problems and markedly reduced growth (not shown). Whilst at birth body weights of *sSURF1*^*−/−*^ piglets were comparable with those of control littermates, they failed to thrive and remained significantly smaller than cloned age-matched wild-type controls and *sSURF1*^*+/−*^ animals (Fig. 2B). Both longer-lived *sSURF1*^*−/−*^ piglets died of sepsis, favoured by dysphagia and by the general critical state of the animals in the last days before death. The infectious agents of which the two piglets died were detected only in these two animals, not in any other piglet housed in the same facility, suggesting a severe debilitation with consequent impaired immune system function.

As for the *sSURF1*^*+/−*^ controls, 14 male and 12 female piglets were generated following the same procedures as for *sSURF1*^*−/−*^, and they appeared phenotypically normal without the clinical signs observed in the *sSURF1*^*−/−*^ animals. Overall, the Kaplan-Meier survival curve showed a highly significant reduction in the lifespan of *sSURF1*^*−/−*^ (survival median 3 days) vs. *sSURF1*^*+/−*^ and wild type control piglets (log-rank test p < 0.001) at 28 days (Fig. 2C). No significant differences resulted from the comparison between survival rates of *sSURF1*^*+/−*^ piglets and wild type controls (log rank test p = 0.264).

Comparison between blood lactate measurements at birth in *sSURF1*^*−/−*^ vs. *sSURF1*^*+/−*^ piglets showed no difference, however strong individual variability was observed among both knockout (n = 12) and control (n = 8) groups (Fig. 2D). In addition, no significant difference was detected between blood lactate level of longer-lived *sSURF1*^*−/−*^ ID498 and age-matched controls (not shown). Standard blood analysis, including TSH, FT3 and FT4, showed no significant differences between *sSURF1*^*−/−*^ animals and controls (not shown).

### 3.5 Biochemical and molecular analysis of *sSURF1*^*−/−*^ tissues

**Fig. 3:**
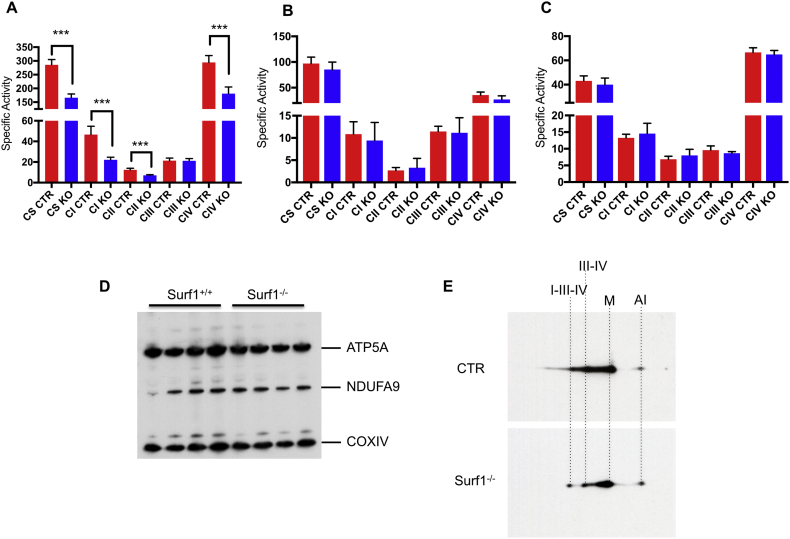
Biochemical characterization of swine SURF1^−/−^ tissues. A–C) MRC specific activities (nmoles/min/mg of proteins) in quadriceps (A) liver (B), and brain (C) of newborn *SURF1*^*−/−*^ piglets. Two-tailed, unpaired, unequal variance Student's *t*-test was used for statistical analysis (***p < 0.005). D) Western blot analysis of subunits of the respiratory chain. E) 2-D BNGE Western-blot of muscle mitochondria from a newborn *SURF1*^*−/−*^ piglet and an age-matched control. Immunovisualization was obtained by using an anti-COI specific antibody. I-III-IV and III-IV are COX-containing supercomplexes. M indicates the COX monomer and AI a commonly detected assembly intermediate.

Spectrophotometric analysis of the MRC activities was performed in tissue homogenates from quadriceps, liver and brain of one-day old *sSURF1*^*−/−*^ animals and age-matched wild-type (WT) controls (Fig. 3A). In addition, we analysed the two longer-lived animals (ID498 and ID525, 80 and 29 days of age, respectively) compared to age-matched controls (not shown). We detected a highly significant decrease of the CS specific activity measured in skeletal muscle homogenates of *sSURF1*^*−/−*^ animals compared to controls, as low as approximately 60% of the control mean (p < 0.0001). Thus, whilst the values of MRC activities normalized to CS did not significantly differ between *sSURF1*^*−/−*^ and control muscle homogenates, the specific activities were significantly reduced for complex I (p = 0.0019), complex II (p = 0.0018) and complex IV (p = 0.0038) (Fig. 3A). Surprisingly, no defects were detected in both liver (Fig. 3B) and brain (Fig. 3C). Western blot immunovisualization confirmed a slight reduction of respiratory chain subunits in skeletal muscle (Fig. 3D). 2D-BNGE showed a reduction in CIV-containing supercomplexes (I-III_2_-IV and III_2_-IV) but no accumulation of assembly intermediates in *SURF1*^*−/−*^ vs. wild type age-matched control mitochondria isolated from skeletal muscle (Fig. 3E). Since SURF1 is a ubiquitous protein and *hSURF1* mutant patients and *Surf1*^*−/−*^ mice show COX deficiency in all tissues and cell types, we analysed COX activity in primary fibroblasts derived from *sSURF1*^*−/−*^ and age matched wild type piglets. Cultured fibroblasts derived from 3 control and 4 *sSURF1*^*−/−*^ animals were analysed at early passages and under age-related stress conditions, i.e. after 15 passages in culture (Supplementary Fig. 4A), but no significant difference was detected with the corresponding controls in either condition. These results were confirmed by histochemical staining for COX (Supplementary Fig. 4B). In addition, no obvious differences were detected in the mitochondrial network (Supplementary Fig. 4B).

**Fig. 4:**
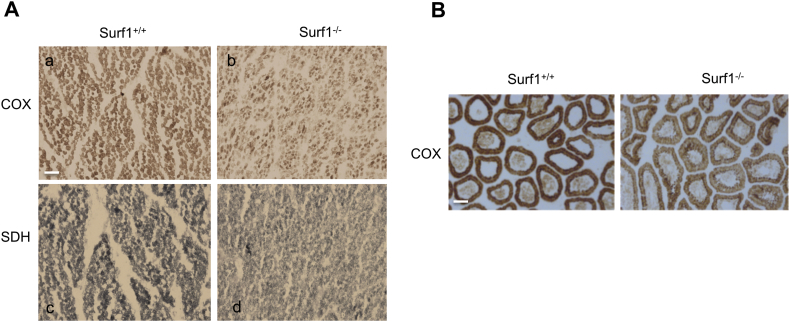
Histochemical findings in swine SURF1^−/−^ model. A) COX (a, b) and SDH (c, d) staining of quadriceps from newborn *SURF1*^*+/+*^ and *SURF1*^*−/−*^ piglets. Note the paler colour in both stainings in *SURF1*^*−/−*^ samples, suggesting a general reduction of MRC activities. B) COX staining of jejunum villi from newborn *SURF1*^*+/+*^ and *SURF1*^*−/−*^ piglets. Note the reduction in COX staining in the *SURF1*^*−/−*^ sample.

Histochemical COX staining in skeletal muscle confirmed a mild reduction in COX reaction in *sSURF1*^*−/−*^ samples compared to controls (n = 8 controls vs. 9 knockout). However 2/9 samples showed no reduced COX staining. No clear differences were detected in the succinate dehydrogenase (SDH) staining in *sSURF1*^*−/−*^ vs. *sSURF1*^*+/+*^ (Fig. 4A), although 2/9 *sSURF1*^*−/−*^ muscles showed that SDH staining was slightly increased compared to the controls. The absence of SDH deficiency may be due to the poor sensitivity of SDH staining, which cannot detect the mild reductions we measured in the spectrophotometric assays. Finally, the same analysis on jejunum samples revealed the presence of villi with pronounced COX deficiency (Fig. 4B). This result could be related to the observed reduced growth of the *sSURF1*^*−/−*^ piglets.

**Fig. 5:**
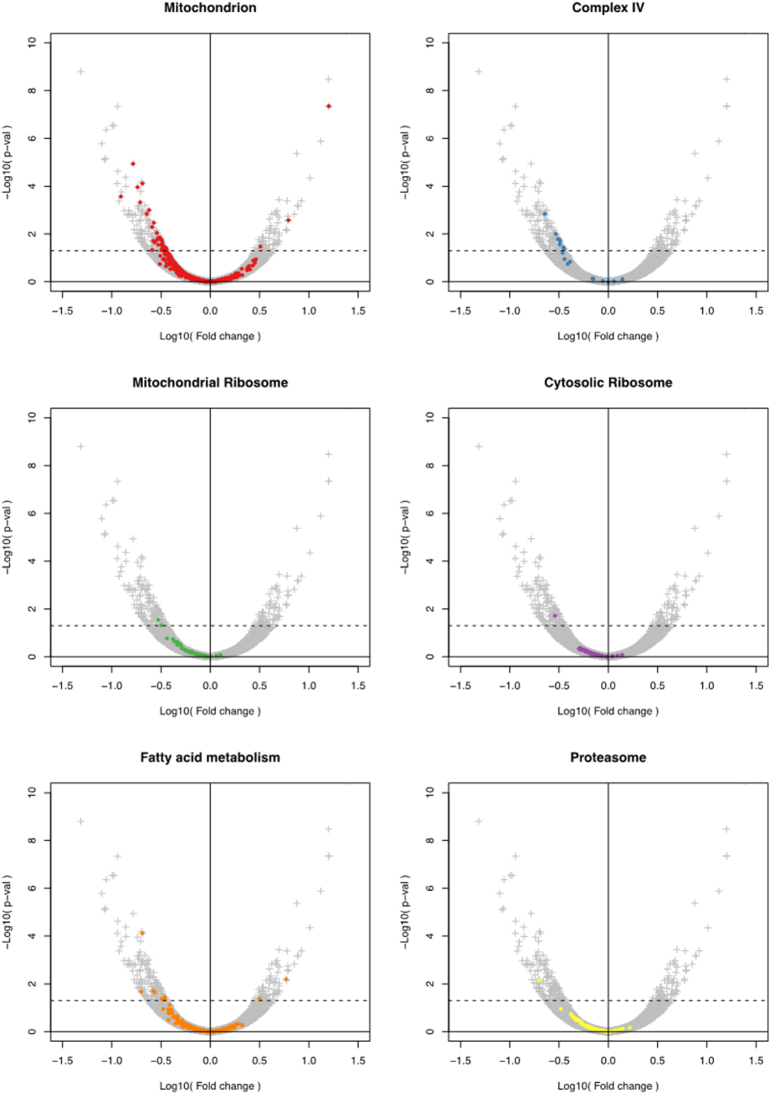
The main pathways affected by SURF1 ablation in pigs. Each volcano plot shows for a gene the fold-change in its expression versus the statistical significance (p-value) of its fold-change between SURF1 ablated pigs (n = 2) and WT pigs (n = 2) (calculated by using DESeq2). Each panel highlights genes of different Gene Ontology terms: mitochondrion (red); complex IV (blue); mitochondrial ribosome (green); cytosolic ribosome (purple); fatty acid metabolism (orange); and proteasome (yellow). Gene sets encoding subunits of the respiratory complexes (only complex IV shown for clarity) or classified according to the Gene Ontology terms “mitochondrial and cytosolic ribosome”, “fatty acid metabolism” and “proteasome” showed a biased distribution towards being under-expressed (calculated by using the non-parametric Mann-Whitney-Wilcoxon two-sample test).

The puzzling discrepancy between the severity of the phenotype of *sSURF1*^*−/−*^ piglets and the lack of specific COX deficiency typical of *hSURF1* mutated patients and *Surf1*^*−/−*^ mice, prompted us to perform an unbiased total mRNA sequencing experiment on skeletal muscle of *sSURF1*^*−/−*^ and WT animals (n = 2/group). A first result was that *sSURF1* mRNAs in genotypically proven *sSURF1*^*−/−*^ animals were indeed carrying the expected mutations (Supplementary Fig. 5). Differential expression of genes was evaluated using DESeq2. About 70 genes were over-expressed and 142 were under-expressed (adjusted p-value <0.05) in *sSURF1*^*−/−*^ vs. WT animals (Supplementary Table S4). Interestingly, several genes encoding subunits of the mitochondrial respiratory chain were present among the under-expressed genes. DAVID functional analysis of these genes indicated that Gene Ontology (GO) terms that were significantly (Benjamini p-value <0.05) over-represented included “cytochrome *c* oxidase activity”, “mitochondrial respiratory chain”, “mitochondrial respiratory chain complex I”, and “mitochondrial respiratory chain complex IV” (not shown), suggesting a role of SURF1 in assembly/stability of supercomplexes, in keeping with the 2D-BNGE experiments. In contrast, for the over-expressed genes there were no GO clusters that were significantly over-represented.

By broadening the functional analysis of the under-expressed genes, including the genes with a fold-change difference of >20% compared to the wild-type (about 3000 genes), GO terms significantly over-represented included “translation”, “mitochondrion”, “oxidative phosphorylation” and “cytosolic large ribosomal subunit” (Supplementary Table S5). To investigate further the differences in expression of the different functional categories of genes, we highlighted various gene sets defined in terms of structural complexes or GO classification on volcano plots of all the differentially expressed genes (Fig. 5). Some gene sets showed a biased distribution towards being under-expressed, including known mitochondrial genes, genes belonging to complex IV, the cytosolic ribosome, the mitochondrial ribosome, or annotated in the Gene Ontology as involved in fatty acid metabolism or the proteasome.

### 3.6 Neuropathological findings

To investigate the presence of abnormalities in the nervous system, we examined four *sSURF1*^*−/−*^ piglets culled at perinatal stage and the two longer-lived *sSURF1*^*−/−*^ piglets (29 d and 80 d of age) and age matched controls. No overt neurodegeneration was detected in H&E stained sections through the entire extension of the central nervous system (CNS); only some cleaved caspase 3 positive neurons were detected in hypoglossal nerve nuclei in the brainstem of the 29 day-old *sSURF1*^*−/−*^ piglet (data not shown).

**Fig. 6:**
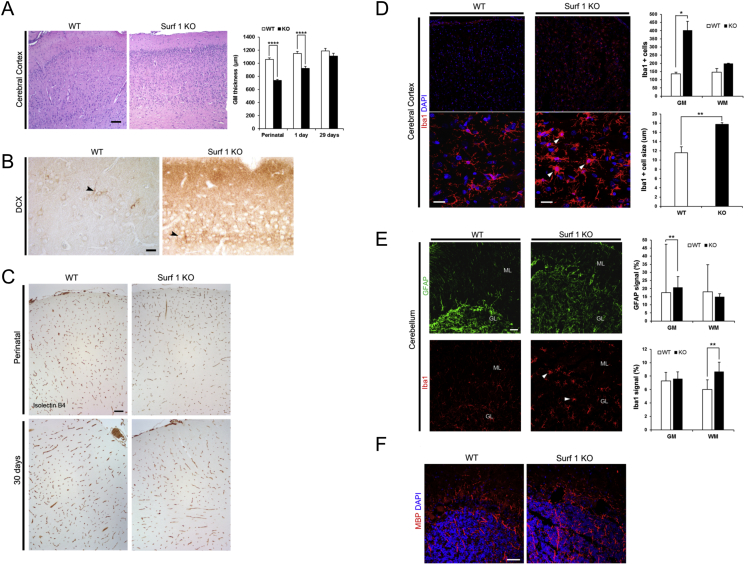
Histological analysis of brain. A) H&E of *SURF1*^*+/+*^ and *SURF1*^*−/−*^ piglets showing unidentifiable layer formation, high cellular density and a significant reduction of cerebral cortex grey matter thickness in newborn *SURF1*^*−/−*^ piglets. Quantification of these parameters are shown in the graph. Data are means ± SEM, unpaired *t*-test, ****p < 0,0001. Scale bar, 100 μm. B) Doublecortin (DCX) staining of some immature neurons in the superficial region of cerebral cortex in newborn *SURF1*^*−/−*^ piglets as compared to age-matched *SURF1*^*+/+*^ animals. Scale bar, 25 μm. C) Vascular proliferation assessment by isolectin B4 staining of endothelial cells. No differences in vasculature in *SURF1*^*+/+*^ and *SURF1*^*−/−*^ brains were detected at all ages analysed. Scale bar, 100 μm D–E) Analysis of the brain of the longer-lived piglets. D) Increased Iba1 staining in *SURF1*^*+/+*^and *SURF1*^*−/−*^ piglets' cerebral cortex indicating gliosis with prominent microgliosis. Graphs showing quantification of the two signals (% of the total area) in grey and white matter areas. Data are means ± SD scale bar, 50 μm. E) Iba1 staining of cerebellum from *SURF1*^*+/+*^ and SURF1^−/−^ animals. Graph shows the number of Iba1 positive cells. Control animal shows resting microglia with a small soma and ramified morphology. In the SURF1^−/−^ litter, activated microglia appears swollen with shorter, less ramified and thicker processes. Graph showing microglia mean size. Data are presented as means ± SD. Scale bar, 20 μm. F) MBP staining in *SURF1*^*+/+*^ and SURF1^−/−^ cerebellar cortex showing comparable myelinated process at 80 days of age. Scale bar, 50 μm.

The most consistent finding in *sSURF1*^*−/−*^ piglets was a significant reduction in the cortical thickness of the cerebrum at early postnatal ages as compared with wild type swine (*sSURF1*^*−/−*^ perinatal: 738 ± 16 μm vs. *sSURF*^*+/−*^: 1057 ± 23 μm; unpaired *t*-test p ≤ 0.0001; *sSURF1*^*−/−*^ 1 day old: 921 ± 31 μm vs. *sSURF*^*+/−*^ 1150 ± 31 μm, unpaired *t*-test p ≤ 0.0001); however, this decrease was not statistically significant in the 29 day old *sSURF1*^*−/−*^ piglet (*sSURF1*^*−/−*^: 1108 ± 43 μm vs. *sSURF*^*+/−*^ 1186 ± 41 μm, unpaired *t*-test p = n.s.; Fig. 6A). Moreover, cortical layering differences were observed in perinatal *sSURF1*^*−/−*^ piglets as compared to controls. In particular, *sSURF1*^*−/−*^ animals showed a higher cellular density and a disorganized cortical structure with several immature neurons, as also revealed by DCX immunostaining (Fig. 6B). No signs of vascular proliferation were detected at all ages analysed by means of Isolectin B4 immunostaining (Fig. 6C). Architecture of basal ganglia, hippocampus, thalamic region and cerebellum were similar to age matched control. GFAP staining revealed a different glial cell distribution pattern between *sSURF1*^*−/−*^ and *sSURF1*^*+/−*^ piglets at all ages analysed. In *sSURF1*^*+/−*^ animals, GFAP was expressed mainly in the white matter (WM) areas whereas *sSURF1*^*−/−*^ brains showed numerous GFAP positive astrocytes in the grey matter (GM) of cerebral and cerebellar cortex, especially around the vessels. Quantification of the signal in the cerebellum confirmed this trend in the longer-lived *sSURF1*^*−/−*^ (Fig. 6D). Iba1 staining pattern was similar in *sSURF1*^*−/−*^ and WT piglets and revealed a more uniform distribution of microglia between white and grey matter areas at perinatal and 29 days of age. Quantification of Iba1 signal in the cerebellum revealed an increase in the 80 days longer-lived *sSURF1*^*−/−*^ piglet (Fig. 6E). The same piglet showed diffuse microgliosis, more prominent in the cerebral cortex grey matter with activated microglial cells showing hypertrophic rounded soma with thickened, shorter and fewer ramified processes as confirmed by quantification (Fig. 6E, lower panel). The analysis of Luxol Fast Blue histological staining and MBP immunohistochemical staining in cortex, basal ganglia, mesencephalon and cerebellum of the older *sSURF1*^*−/−*^ piglet revealed normal myelinating process (Fig. 6F).

## 4 Discussion

Mouse models are valuable tools to unravel the molecular and biochemical mechanisms underpinning mitochondrial diseases, but they often fail to develop the qualitative and/or quantitative clinical features of the human pathology. *Surf1*^*−/−*^ mice are a striking example of this, as they display partial COX deficiency, but hardly any pathological phenotype. Conversely, in LS patients, SURF1 ablation causes a devastating, early-onset, invariably fatal encephalopathy associated with a typical isolated COX deficiency. We thus decided to produce the first *sSURF1*^*−/−*^ pig model. To this aim, we managed to sequence the whole *sSURF1* gene and annotated all the 9 exons (only exons 3–5 were previously annotated on public databases). We disrupted the *sSURF1* gene using two alternative methods, TALENs and CRISPRs, in primary porcine fibroblast cell lines, and used them for high efficiency SCNT. The pathological phenotypes observed in the two knock-out models of *sSURF1*^*−/−*^ piglets were remarkably similar, independently of the nuclease used and the specific gene disruption obtained by the two approaches, indicating that it was only related to the absence of *sSURF1* and ruling out the possibility that it could be due to off-target effects. Notably, *sSURF1*^*+/−*^ piglets (both males and females) that were generated using the same reagents and procedures as *sSURF1*^*−/−*^ littermates did not show any pathological sign and did not differ significantly from *sSURF1*^*+/+*^ controls. Moreover, the older *sSURF1*^*+/−*^ sow was demonstrated to be fertile as a pregnancy was successfully established using a WT boar.

The clinical phenotype associated with the ablation of *sSURF1* was extremely severe and characterized by growth retardation/arrest, failure to thrive and persistent tremors, supporting a fundamental role of SURF1 in post-natal survival of the pig. The survival of the piglets could only be prolonged a few weeks by rising them in intensive care and with artificial feeding. Surprisingly, however, a multiple defect of the specific activities of complexes I, II and IV was detected in *sSURF1*^*−/−*^ skeletal muscle, associated with marked decrease of CS activity, whereas no MRC defect was measured in cultured fibroblasts. A puzzling feature of the *sSURF1*^*−/−*^ pigs was the variability observed in COX and SDH histochemical reactions. Nevertheless, the biochemical quantification of COX and CII specific activities showed a significant decrease in *sSURF1*^*−/−*^ muscle samples compared to controls, whereas no significant differences were detected in other tissues (e.g. liver, brain, and cultured fibroblasts). This is also an unexplained discrepancy of the *sSURF1*^*−/−*^ model, compared to other mammalian organisms, including mouse and human, which are characterized by generalized COX deficiency, detected in all tissues and cell types examined.

A role for SURF1 in COX assembly and/or stability has been demonstrated in several organisms where the absence of SURF1 leads to COX deficiency and accumulation of COX assembly intermediates [37]. In yeast, a role for the SURF1 ortholog, SHY1, in the hemilation of COX1 has been proposed, whereas in humans SURF1 has been suggested to be involved in the incorporation of COX2 in nascent complex IV [38]. In other species, notably in *Drosophila melanogaster*, constitutive knockdown of *d.SURF1* is associated with poor somatic growth of larvae, and developmental arrest leading to death at the pre-pupal stage, with modest but significant defects of all the MRC activities [39]. This resembles the condition observed in our *sSURF1*^*−/−*^ animals. Interestingly, when the *dSURF1* knockdown is restricted to the cephalic segment of the adult fly, then a clear cut, isolated COX defect is detected [39]. The exact role of SURF1 in COX assembly in mammals remains, however, unclear. Taken together, these observations suggest that SURF1 may play a role in the stabilization/activity of the MRC complexes, possibly in a species and developmental specific manner. Accordingly, it has been recently shown that COX accumulates less in SC in mouse than in humans and that COX is more stable in *Surf1*^*−/−*^ mice than in LS^SURF1^ patients [37]. In case of *sSURF1*, its complete constitutive ablation severely impairs the somatic development and overall survival of the neonatal or suckling piglets, associated with reduced activity of COX as well as other mitochondrial enzymes, including MRC complexes and CS, in skeletal muscle. Indeed, our RNAseq analysis showed that the expression of several transcripts associated with the respiratory chain were down regulated in *sSURF1*^*−/−*^ vs. *sSURF1*^*+/+*^ animals. In addition, other pathways, including translation and proteome related-genes were significantly affected, suggesting *sSURF1* ablation interferes with several pathways, although their contribution to the phenotype is currently unknown.

The most striking clinical phenotype observed in our *sSURF1*^*−/−*^ piglets is reduced/arrest of somatic growth, which is not surprising if one considers that weight gain, mainly due to massive expansion in muscle and fat tissues, is possibly the most prominent feature of farmed pre-pubertal swine. In contrast with what is observed in humans, the absence of *sSURF1* in pigs resulted in modest neuropathological changes at early postnatal ages, consisting of differences in cortical architecture associated with the presence of immature still migrating DCX positive neurons and numerous astroglial cells. A consistent feature was the presence of microgliosis especially within the cerebral cortex in the longer-surviving *sSURF1*^*−/−*^ animal, a feature that is also reported in LS associated pathology. Although an inter-individual heterogeneity should be considered, a delayed or altered cortical development of *sSURF1*^*−/−*^ swine could not be excluded but these features are modest compared to the dramatic somatic growth arrest, and certainly marginal in comparison to the cerebral lesions hallmarking the human condition. It has to be noted that in the clinical cases of LS (as in any clinical situation) the complete genetic background is not known, thus the presence of compensatory mechanisms or mutation(s) in other genes can change the course of the syndrome. For example, a recent paper identified healthy individuals resilient to highly penetrant forms of genetic childhood disorders [40]. Therefore, the information coming from the study of each animal model of the same pathology (e.g. LS) does not or cannot necessarily replicate exactly the human condition, but can be useful to investigate different metabolic and/or physiologic pathways involved in the onset of the pathology of interest. In addition, in humans the brain/body mass ratio is clearly different from that of other animals and this could explain why in humans the cerebral lesions prevail, whereas in the pig the prevailing tissues affected are the intestine and skeletal muscles, whose failure brings the animals to death with no major cerebral lesions. In conclusion, we describe a dramatic clinical phenotype in pigs associated with the ablation of *sSURF1*, which is not accompanied by the severe biochemical and structural defect of COX typical of human LS^SURF1^. The mechanistic aspects of SURF1 in COX biogenesis and, possibly, other mitochondrial pathways are still poorly understood. The creation of constitutive, developmentally regulated models of *SURF1* ablation in different species could help elucidate the physiological role(s) of this elusive protein in mitochondrial and cell metabolism, and clarify the complex pathogenesis of the *SURF1*-associated human disease.

The following are the supplementary data related to this article.

Supplementary material Image 1

Supplementary Table S4 Differential expression of genes evaluated using DESeq2. Supplementary Table S4

Supplementary Table S5 The most enriched GO terms in the under- and over-expressed genes as assessed by the DAVID Functional Annotation Tool. Supplementary Table S5

## Transparency document

Transparency document Image 2
