# Supplementary material for: SURF1 knockout cloned pigs: Early onset of a severe lethal phenotype
Source: Biochim Biophys Acta. 2018 Jun;1864(6Part A):2131–42. doi: 10.1016/j.bbadis.2018.03.021 (PMC6018622; doi:10.1016/j.bbadis.2018.03.021)
Supplement: Supplementary file 1 — Supplementary material [file mmc1.docx]

**SUPPLEMENTARY MATERIAL**

**Supplementary Methods**

**Sequencing of swine Surf1 gene**

As for PCRs, only the amplification conditions of the whole *sSurf1* gene PCR are here specified. PCR reaction was performed with S1Flong and S1Rlong primers. Amplification was performed in 12.5 μL reaction volume (0.4 mM dNTPs, 0.8 µM each primer, 0.05 U/µl LA-Taq in GCI Buffer, Nuclease-free H_2_O to volume) using a touchdown protocol as follows: 94°Cx2’, 8 cycles: 94°Cx30”, 72°Cx30”-1°C/cycle, 72°Cx5’ and 32 cycles: 94°Cx30”, 65°Cx30”, 72°Cx5’+5”/cycle, followed by a final elongation at 72°Cx7’. The specific PCR product is 4582bp.

As for the RT-PCR, the reaction was performed with S1F and S1R primers. Amplification was performed in 12.5 μL reaction volume (0.4 mM dNTPs, 0.8 µM each primer, 0.05 U/µl LA-Taq in GCI Buffer, Nuclease-free H_2_O to volume) and thermal cycler programme was a touchdown cycle characterized by 94°Cx2’ as initial denaturation, 3 cycles: 94°Cx30”, 72°Cx30”-1°C/cycle, 72°Cx1’ and 37 cycles: 94°Cx30”, 70°Cx30”, 72°Cx1’, followed by a final elongation at 72°Cx7’. The specific PCR product is 921bp.

The complete sequence of *Sus scrofa* Surf1 gene we obtained from our wild type male cell line ID6639 has been deposited, accession number BankIt2034014 Surf1     MF535518.

**Homologous Recombination (HR) Vector construction**

Genomic DNA was extracted from pig fibroblasts and a PCR product covering the *Surf1* region between exon 2 and exon 5 was cloned into the pSMART®GC HK vector (Sigma, UK). An inside-out PCR was performed using primers with adapted ends (NotI restriction enzyme cutting site was included) flanking exon 3 in order to delete 106 bp of exon 3. The resulting linear construct was re-ligated by Gibson assembly (New England Biolabs, Ipswich, MA). Finally, a floxable puromycin resistance cassette was cloned into the HR vector thanks to the newly created NotI site in the intron. This vector targets swine *Surf1* exon 3 by exploiting homology regions upstream (exon 2) and downstream (exon 4 and exon 5) in order to exchange wild type exon 3 with the 106 bp-deleted version of exon 3 present in this vector and thus making a null allele at the insertion site (Figure 1).

**Animal Genotyping**

Genomic DNA was extracted from ear or tail biopsies (from stillborn or alive piglets, respectively) by overnight incubation at 55°C with cell lysis solution (100 mM Tris HCl pH 8.3, 5 mM EDTA pH 8.1, 0.2% SDS, 200 mM NaCl) supplemented with 100 μg of Proteinase K/mL (Macherey-Nagel, Germany) (1), re-suspended in 500 μL of TE buffer and quantified using the Qubit system (Invitrogen, S. Giuliano Milanese, Italy). The same PCRs used for colony screening (Ex2F + PuroF2-R and S1T Forward + S1T Reverse) were applied for piglets genotyping and piglet/colony correspondence was confirmed. The integration of HR vector was screened with primer Ex2Fw and PuroF2-R. PCR reaction for sequencing was performed with S1T Fw and S1T Rv primers.

**Isolation of Mitochondria**

Mitochondria were extracted by differential centrifugation using medium AT (0.075 M sucrose, 0.225 M mannitol, 1 mM ethylene glycol tetra acetic acid EGTA, 0.01% bovine serum albumin BSA, pH 7.4) and finally re-suspended in the appropriate volume of MAITE medium (25 mM sucrose, 75 mM sorbitol, 100 mM KCl, 0.05 mM EDTA, 5 mM MgCl_2_, 10 mM Tris–HCl, 10 mM H_3_PO_4_, pH 7.4).

**Western blot, Blue Native and In-Gel activity analysis**

Western blot analysis, performed on electroblotted denaturing sodium-dodecyl sulphate polyacrylamide gel electrophoresis (SDS–PAGE), and two-dimension blue native gel electrophoresis (2D-BNGE) analysis were performed as previously described (2). Approximately 100 µg of non-collagenous proteins were used for each sample in SDS-PAGE and 20µg of isolated mitochondria in 2D-BNGE. Chemiluminescence-based immunostaining (ECL kit, Amersham) was performed using the following antibodies: anti Surf1 (abcam ab110256); anti COX I (Molecular Probes, Eugene, OR, USA) and anti COX IV (Mitosciences LLC, Eugene, OR, USA). In Gel Activity analysis was performed as described in reference (3).

**Supplementary Table 1. Sequences of the TALENs repeat-variable di-residue (RVDs) used in the study**

| **TALEN name** | **Amino-acidic sequence** |
| --- | --- |
| GT-EN-12532-01L (Tal01L) | HDNGHDHDNGNINNNNNNHDNNNNNNHDHDNGNN |
| GT-EN-12532-01R (Tal01R) | NGHDNINNHDNGNNNININNNINNHDNGNNHDHD |
| GT-EN-12532-02L (Tal02L) | NGNGHDNGHDHDNINNNGNNNNNGNGNGHDNGNI |
| GT-EN-12532-02R (Tal02R) | HDHDHDHDNINNNNHDHDNINININNNNHDNINN |
| GT-EN-12532-03L (Tal03L) | NINNNNNGHDHDNINNHDNNNGHDNNNNNININN |
| GT-EN-12532-03R (Tal03R) | NNNNNIHDNGHDHDNINNNGNGHDNNNNHDNNNI |

**Supplementary Table 2. Sequences of CRISPR/Cas9 gRNA**

| **gRNA Name** | **Sequence 5'-3'** |
| --- | --- |
| ♯Guide1Fw | TGTATGAGACCACACGATGATGCCTTTCTCCAG |
| ♯Guide1Rv | AAACCTGGAGAAAGGCATCATCGTGGTCTCA |
| ♯ Guide2Fw | TGTATGAGACCACGGCATCATCGTGCGCTTTTG |
| ♯Guide2Rv | AAACCAAAAGCGCACGATGATGCCGTGGTCTCA |

**Supplementary Table 3. Sequences of the primers used throughout the study**

| **Oligo name** | **Sequence 5'-3'** |
| --- | --- |
| S1Flong | ATGGCGGCGCGGTGGCTGGGGC |
| S1Rlong | TGAGGCGGCCTCCTGCGGTGTGA |
| S1T Fw | GAGCCTCCACTTCTTTGTCC |
| S1T Rv | ﻿CCACATACACTCACTCTGCGG |
| Ex2Fw | CGTCGGGAGGAGCGTCCTTG |
| Ex4Rv | GCAGCGGGATGGGCTCAGCC |
| PuroF2-R | ﻿CCAGCTCATTCCTCCCACTCATGA |
| S1F | ATGGCGGCGCGGTGGCTGGG |
| S1R | TCACACCGCAGGAGGCCGCC |
| S1Flong | ATGGCGGCGCGGTGGCTGGGGC |
| S1R1 | CCCCGTTGGAGGACAGACCA |
| S1R2 | CGTGGGGATGGTGAGGCTGA |
| S1R3 | CGCCCACGTTGTCACTCCCT |
| S1R4 | GGGGCACTTTGACCACTCCA |
| S1R5 | CGCCGAACTGGAGTCCAGGA |
| S1R6 | CCTTTGGCCTGGGGACATGG |

**Supplementary figures**

**Supplementary Figure 1. Characterization of *sSURF1*gene**

**
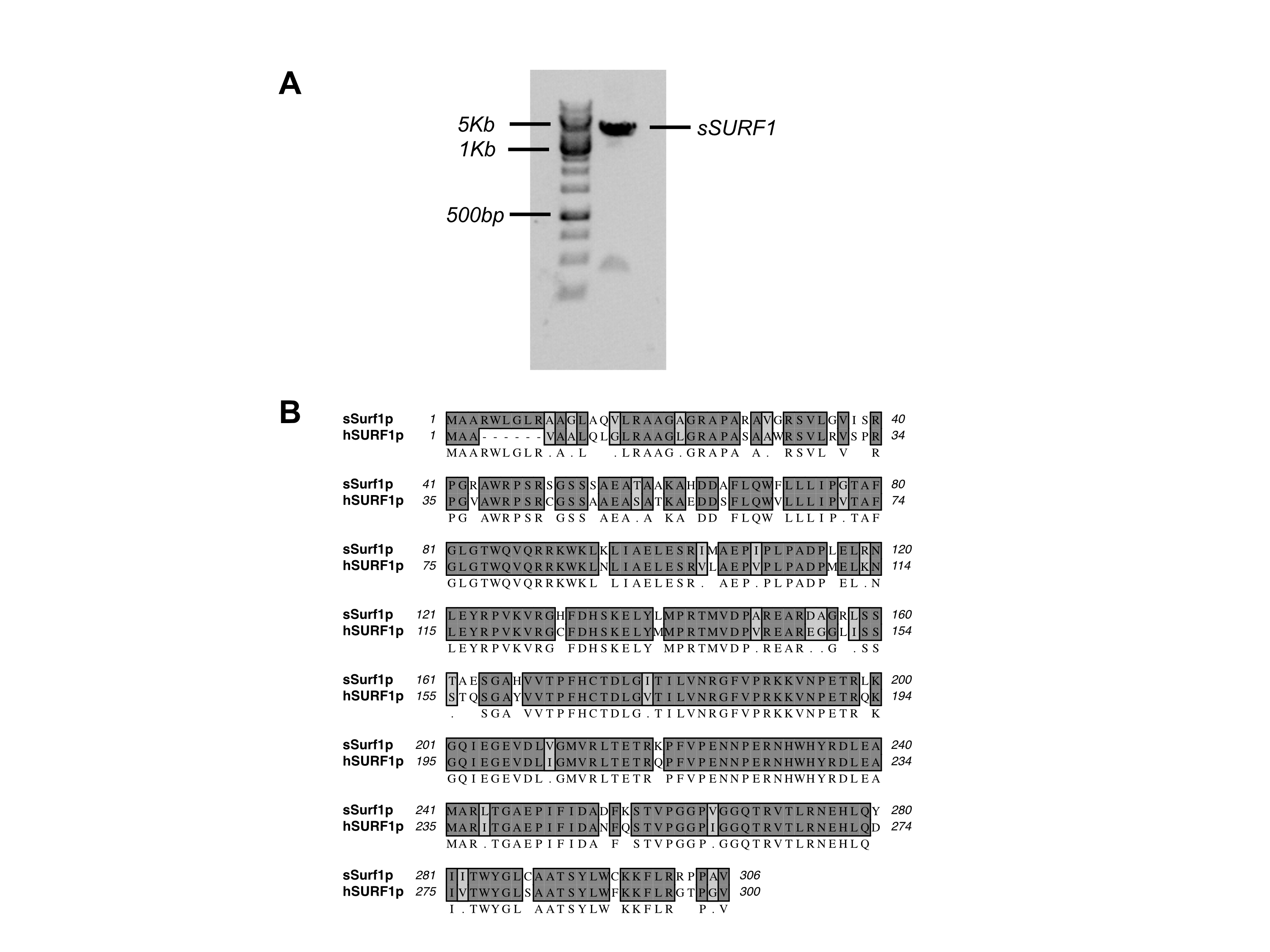
**

A) Amplification of the full-length genomic *sSURF1* gene

B) Clustal Omega alignment of hSURF1, A0A0B8RZZ9 sequence, annotated as swine SURF1 in NCBI database, and the sSURF1 protein determined by us.

**Supplementary Figure 2. Alignments of the NHEJ allele of the two founders piglets generated through TALENs (A) and CRISPRs (B) with the wild-type.**


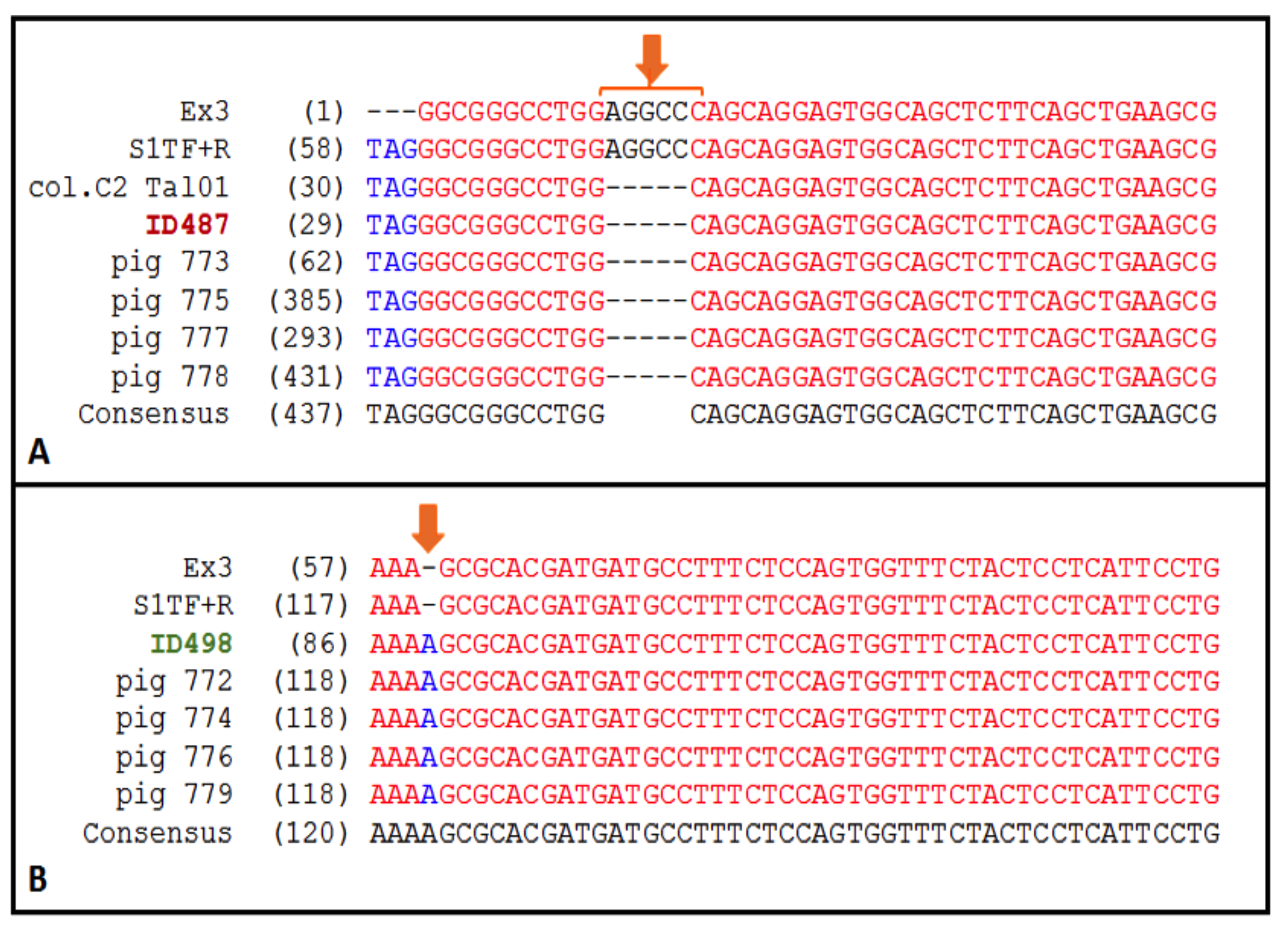


A) 5-bp deletion in the NHEJ allele from colony C2Tal01 and 5 pigs generated by TALENs; among them, piglet ID487, whose cells were used for further re-cloning experiments of TALENs-derived SURF1^-/-^ animals throughout the paper including ID773, 775, 777, 778.

B) 1-bp insertion in the NHEJ allele from piglet ID498 (the only SURF1^-/-^ obtained with CRISPRs technology) and 4 heterozygous piglets (ID 772, 774, 776, 779) generated by CRISPR/Cas9.

**Supplementary Figure 3. Two-step strategy for disrupting *sSURF1* using CRISPR/Cas9 system and generating *SURF1^-/-^* pigs**

**Supplementary Figure 4. Characterization of porcine *Surf1^-/-^* fibroblasts.**

A) COX specific activity (ng/min/mg of protein) in *Surf^+/+^* (n=3) and *Surf1^-/-^* (n=4) fibroblasts cell lines derived from corresponding newborn piglets. Data are expressed as mean± SEM.

B) Upper panel: COX staining; lower panel: mitotracker staining.

**Supplementary Figure 5. SURF1 expression analysis by RNAseq.**


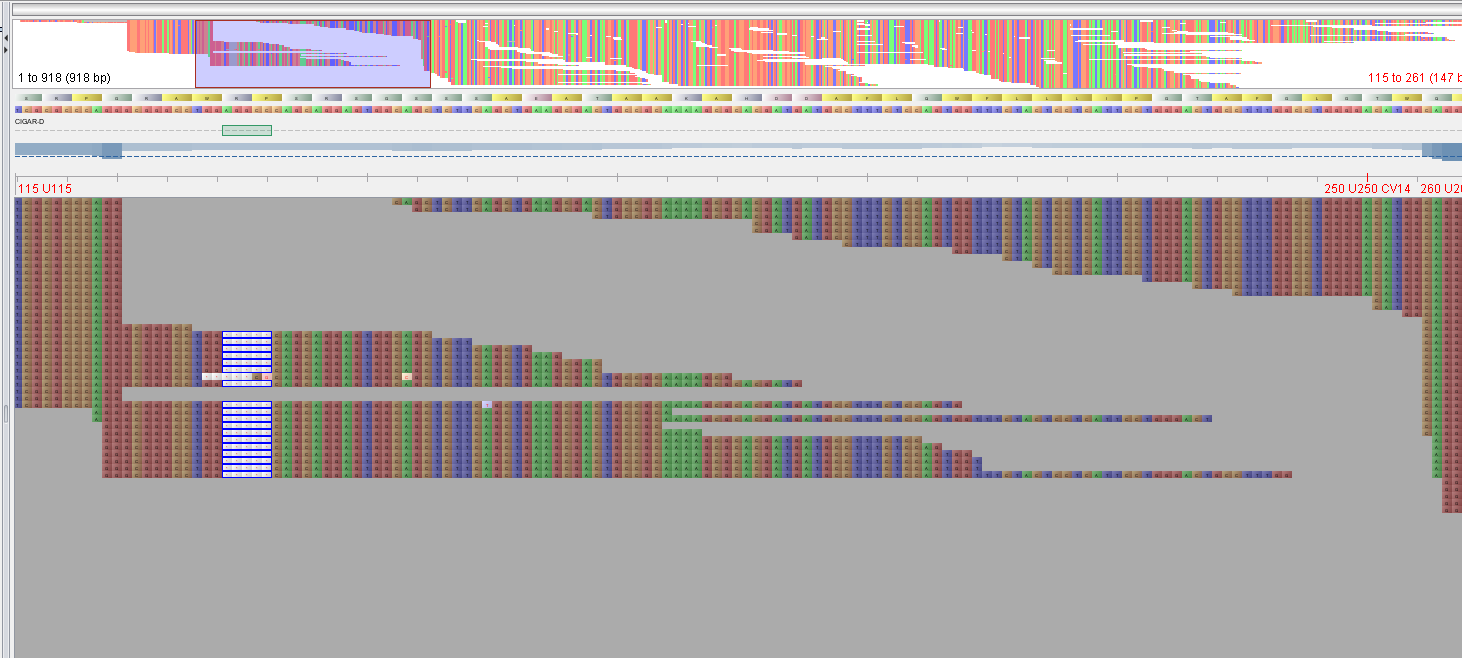


5-bp deletion

**KO**


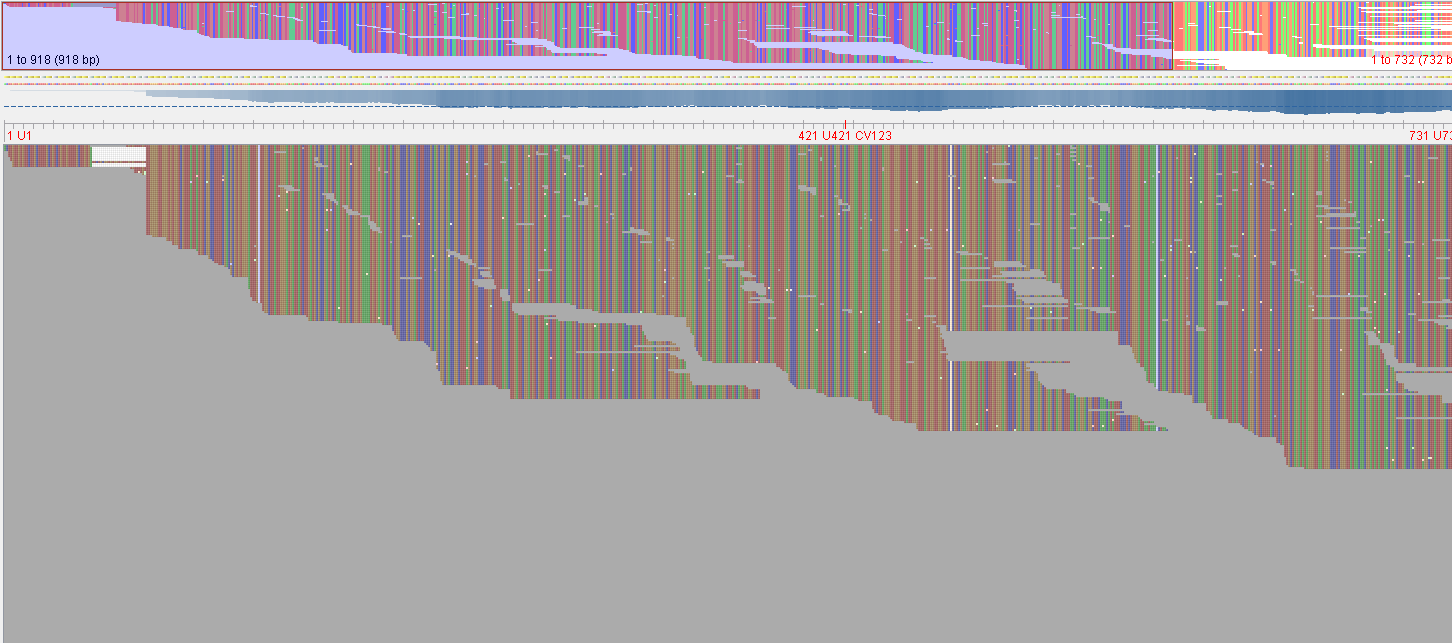

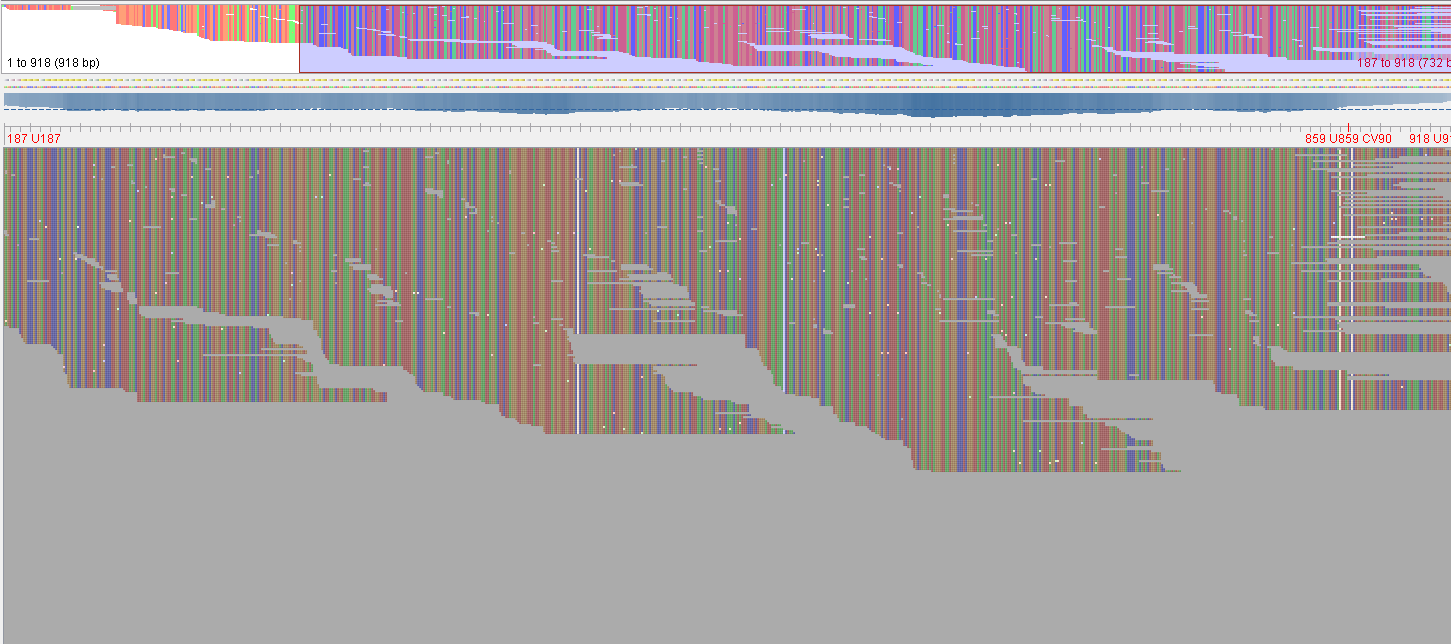


**CTR**

Analysis of Surf1 isoforms detected by RNAseq in *SURF1^-/-^* (upper panel) and *SURF^+/+^* (bottom panel) muscles by BWA-MEM analysis. BWA-MEM does not require one end-to-end alignment for anchoring, but allows for partial alignments using split-read mapping. In this way, each read can be mapped to two separate locations because of possible structural variation (in this case the deletion of 106 nt displayed as an empty part in the figure). The red brackets indicate the left and right read boundaries of otherwise continues reads.

Note that in the *SURF1^-/-^* samples only the recombinant alleles, carrying the 106bp deletion and the 5 bp deletion, are detected, as expected as these animals were generated using the TALENs (see also the text for details).

References

1 Sambrook, J., Fritsch, E.F. and Maniatis, T. (1989) *Molecular cloning*. Cold spring harbor laboratory press New York.

2 Tiranti, V., Jaksch, M., Hofmann, S., Galimberti, C., Hoertnagel, K., Lulli, L., Freisinger, P., Bindoff, L., Gerbitz, K.D., Comi, G.P. *et al.* (1999) Loss-of-function mutations of SURF-1 are specifically associated with Leigh syndrome with cytochrome c oxidase deficiency. *Annals of Neurology*, **46**, 161-166.

3 Calvaruso, M.A., Smeitink, J. and Nijtmans, L. (2008) Electrophoresis techniques to investigate defects in oxidative phosphorylation. *Methods*, **46**, 281-287.
