# Supplementary material for: SURF1 knockout cloned pigs: Early onset of a severe lethal phenotype
Source: Biochim Biophys Acta. 2018 Jun;1864(6Part A):2131–42. doi: 10.1016/j.bbadis.2018.03.021 (PMC6018622; doi:10.1016/j.bbadis.2018.03.021)
Supplement: Supplementary Table S5 — The most enriched GO terms in the under- and over-expressed genes as assessed by the DAVID Functional Annotation Tool. [file mmc3.docx]

**Supplementary Table S5**: The most enriched GO terms in the under- and over-expressed genes as assessed by the DAVID Functional Annotation Tool.

| GO Category | Under/Over-expressed | Number of genes | p-val |
| --- | --- | --- | --- |
| Mitochondrion | Under | 918 | 2 x 10^-16^ |
| Ribosome | Under | 174 | 2 x 10^-16^ |
| Proteasome complex | Under | 58 | 4 x 10^-8^ |
| Fatty acid metabolism | Under | 234 | 2 x 10^-8^ |
| Apoptotic process | Under | 1207 | 4 x 10^-6^ |
| Transcription, DNA-template | Over | 2141 | 1 x 10^-5^ |
| Glycogen metabolic process | Under | 66 | 2 x 10^-4^ |
| Autophagy | Under | 337 | 3 x 10^-4^ |
| Response to oxidative stress | Under | 259 | 5 x 10^-4^ |
| Canonical glycolysis | Under | 23 | 1 x 10^-3^ |
